# The Effects of Outdoor Activity on Patient-Reported Visual Outcomes Under Perioperative Management Using Cyclosporine and Rebamipide

**DOI:** 10.3390/diagnostics15131629

**Published:** 2025-06-26

**Authors:** Chung Min Lee, Nahee Kim, Min Young Kim, Nahyun Park, Yea Eun Lee, Jeewon Han, Hayoung Lee, Kyu Sang Eah, Yoo Young Jeon, Ho Seok Chung, Jae Yong Kim, Hun Lee

**Affiliations:** 1 Department of Ophthalmology, Asan Medical Center, University of Ulsan College of Medicine, Seoul 05505, Republic of Korea; chungminlee1215@gmail.com (C.M.L.); laurenpark66@gmail.com (N.P.); yeaeun812@gmail.com (Y.E.L.); jenny4132@naver.com (J.H.); glory2822@naver.com (H.L.); kseah0124@gmail.com (K.S.E.); cheese_sauce@naver.com (Y.Y.J.); chunghoseok@gmail.com (H.S.C.); jykim2311@amc.seoul.kr (J.Y.K.); 2 School of Medicine, Ewha Womans University, Seoul 03760, Republic of Korea; angel_nahee@naver.com (N.K.); kmc1369@naver.com (M.Y.K.); 3 Department of Ophthalmology, Asan Medical Institute of Convergence Science and Technology (AMIST), University of Ulsan College of Medicine, Seoul 05505, Republic of Korea; 4 Department of Ophthalmology, Brain Korea 21 Project, University of Ulsan College of Medicine, Seoul 05505, Republic of Korea; 5 Center for Cell Therapy, Asan Medical Center, Seoul 05505, Republic of Korea

**Keywords:** cataract surgery, Cataract-related Visual Function Questionnaire (CVFQ), outdoor activity, patient-reported outcomes, patient satisfaction

## Abstract

**Background/Objectives**: Visual recovery after cataract surgery may be affected by lifestyle and environmental factors. This study aimed to investigate the association between daily outdoor activity and patient-reported visual outcomes after cataract surgery under perioperative management using cyclosporine and rebamipide. **Methods**: This retrospective study included 90 eyes from patients who underwent standardized cataract surgery with implantation of TECNIS Eyhance intraocular lenses. Patients were divided into two groups based on their average daily outdoor activity during the first postoperative month: Group 1 (≤3 h/day) and Group 2 (>3 h/day). Postoperative assessments included Cataract-related Visual Function Questionnaire (CVFQ) scores, uncorrected and corrected distance visual acuity, and ocular surface parameters such as tear breakup time, Oxford score, SICCA score, and OSDI. **Results**: Group 1 reported significantly higher CVFQ scores for daytime (*p* = 0.020) and night-time driving (*p* = 0.037) compared to Group 2, indicating lower levels of visual discomfort in these tasks. No significant differences were observed between the groups for other CVFQ subcategories or ocular surface parameters. **Conclusions**: Except for driving-related discomfort, no significant differences were found in CVFQ scores between the groups. No difference in ocular surface parameters can be attributed to the perioperative management using cyclosporine and rebamipide. CVFQ may be a useful tool for assessing subjective visual outcomes after cataract surgery.

## 1. Introduction

Cataract surgery is one of the most frequently performed procedures in ophthalmology, with generally favorable outcomes in visual acuity and patient satisfaction [1,2]. A recovery period of approximately one month is commonly recommended to facilitate proper wound healing and visual adaptation [3]. During this period, patients are advised to avoid ocular irritants and adhere to protective behaviors, such as wearing sunglasses during outdoor exposure. However, specific guidelines on activity restrictions—particularly regarding the duration and extent of outdoor activity, exercise, and driving—remain inconsistent and are not well established [4]. A recent comparative review identified considerable variation among postoperative instructions provided by eye care institutions, especially concerning the timing for resuming routine or strenuous activities [5]. These inconsistencies reflect a lack of empirical evidence supporting current recommendations.

In recent years, the incorporation of patient-reported outcomes has become essential in evaluating surgical success, complementing traditional objective metrics such as visual acuity and defocus curves [6]. Various standardized questionnaires have been developed to assess visual function and ocular discomfort. Notably, the Standard Patient Evaluation of Eye Dryness (SPEED) questionnaire and the 25-Item National Eye Institute Visual Function Questionnaire (NEI VFQ-25) are widely used internationally. However, in Korea, these tools have been limited to translated versions and are not specific to cataract-related visual function [7]. To address this gap, the Cataract Visual Function Questionnaire (CVFQ) was recently introduced in Korean clinical settings. CVFQ enables the structured, subcategory-specific assessment of postoperative visual symptoms and task-related discomfort [8].

Given the absence of standardized activity guidelines after cataract surgery and the limited availability of culturally adapted tools for subjective visual assessment in Korea, there is a need for research that evaluates lifestyle factors, such as outdoor activity, in relation to visual recovery. This study aimed to investigate the association between daily outdoor activity levels during the early postoperative period and patient-reported visual outcomes using the CVFQ. Additionally, the study sought to assess the clinical applicability of the Korean version of the CVFQ in cataract patients.

## 2. Materials and Methods

This retrospective, comparative study was conducted at Asan Medical Center, Seoul, Republic of Korea. The study adhered to the tenets of the Declaration of Helsinki and was approved by the Institutional Review Board of Asan Medical Center (IRB No. 20241083). Informed consent was waived due to the retrospective nature of the study. The dataset included patients who underwent standardized cataract surgery with implantation of the TECNIS Eyhance intraocular lens (ICB00, Johnson & Johnson Vision, Santa Ana, CA, USA).

Eligible eyes met the following inclusion criteria: (1) postoperative corrected distance visual acuity (CDVA) of ≥6/12, (2) an emmetropic refractive target, and (3) a minimum of one month of follow-up without data loss. Exclusion criteria were a history of prior ocular surgery, the presence of ocular comorbidities, or the use of medications that could influence visual outcomes. A total of 90 eyes from 90 patients were analyzed. Patients were categorized into two groups according to their average daily outdoor activity during the first postoperative month: Group 1 (≤3 h/day, *n* = 57) and Group 2 (>3 h/day, *n* = 33). This 3-h threshold was based on a previous report indicating an average outdoor activity time of approximately 3.24 h/day in the Korean population [9], as well as data from the Multinational Time Use Study showing that individuals aged ≥50 years spend approximately 221–226 min outdoors daily.

Preoperative and postoperative evaluations included Uncorrected Distance Visual Acuity (UCVA), CDVA, Intraocular Pressure, manifest refraction, and autokeratometry (Tomey, Nagoya, Japan). Postoperative ocular surface assessments included tear breakup time (TBUT), corneal staining graded using both the Oxford system and the Sjögren’s International Collaborative Clinical Alliance (SICCA) scale, and the Ocular Surface Disease Index (OSDI) [10,11]. The OSDI consists of 12 items across three subdomains: ocular symptoms (five items), vision-related function (four items), and environmental triggers (three items). Each item is rated on a 5-point scale (0 to 4), and the total score ranges from 0 to 100, with higher scores indicating greater ocular surface disease severity [12].

Postoperative ocular discomfort was evaluated using the Numerical Rating Scale (NRS), in which patients rate discomfort on a scale from 0 (no discomfort) to 10 (worst possible discomfort). While the NRS is primarily used for assessing acute and chronic pain, it is also suitable for measuring subjective discomfort in ophthalmic settings [13]. Patients were asked to estimate their average daily outdoor activity time during the first postoperative month at the follow-up visit, choosing from predefined categories corresponding to Group 1 or Group 2. Outdoor activities included walking, commuting, and driving, although the exact duration of each activity type was not separately recorded.

All patients received a standardized perioperative regimen consisting of 0.1% cyclosporine (Santen, Osaka, Japan) once daily and 2% rebamipide (Kukje Pharma, Seoul, Korea) four times daily for two weeks preoperatively and one month postoperatively. Additionally, 1.5% levofloxacin (Santen) and prednisolone acetate (Allergan, Irvine, CA, USA) were prescribed four times daily during the first postoperative month.

Subjective visual function and satisfaction were assessed using the Cataract Visual Function Questionnaire (CVFQ), a validated instrument developed specifically for Korean cataract patients. The CVFQ was designed based on existing questionnaires, including the Nine-item Short Form of the Catquest Questionnaire [14], Visual Function Index-14 [15], and Type-Specific Patient Evaluation of Cataract Surgery [16]. To ensure cultural relevance, the CVFQ incorporates items such as reading mobile device text and television subtitles. It comprises 15 items across five subdomains: overall visual quality, overall visual function, distance vision, near vision, and glare-related visual function. Each item is initially rated on a 5-point scale (0 to 4), where 0 accounts for “not sure”, and higher scores indicate greater satisfaction. In accordance with the original scoring structure of the CVFQ, responses are recorded onto a 100-point scale, with values of 0, 1, 2, 3, and 4 converted to 0, 25, 50, 75, and 100, respectively. Each subdomain score is calculated as the arithmetic mean of the recoded item scores within that category, and the total CVFQ score is obtained by averaging the five subdomain scores. Consequently, the final score ranges from 25 to 100, with higher scores reflecting better visual function and greater patient satisfaction. The structure and content of the CVFQ are summarized in Table S1.

All statistical analyses were performed using RStudio (Posit, version 4.4.1, Boston, MA, USA). The normality of continuous variables was assessed using the Shapiro–Wilk test. Depending on data distribution, either the *t*-test or the Mann–Whitney U test was used to compare continuous variables between groups. For categorical variables, such as sex, the chi-square test was used. A two-sided *p*-value of <0.05 was considered statistically significant.

## 3. Results

### 3.1. Patient Characteristics and Biometric Parameters

The demographic and biometric characteristics of the two groups are summarized in Table 1. The mean age was 68.74 ± 12.74 years in Group 1 and 64.15 ± 13.02 years in Group 2, with no statistically significant difference (*p* = 0.110). The postoperative corrected distance visual acuity was 0.92 ± 0.12 in Group 1 and 0.91 ± 0.11 in Group 2 (*p* = 0.593). The mean postoperative keratometry was 43.86 ± 2.02 D and 43.85 ± 1.87 D in Groups 1 and 2, respectively (*p* = 0.981). The postoperative Spherical Equivalent was −0.68 ± 0.98 D in Group 1 and −0.73 ± 0.74 D in Group 2 (*p* = 0.287).

### 3.2. Ocular Surface Parameters and Symptom Scores

The ocular surface parameters and subjective symptom scores are presented in Table 2. The mean tear breakup time was 4.63 ± 1.40 s in Group 1 and 4.33 ± 1.80 s in Group 2 (*p* = 0.452). No significant differences were observed between groups in corneal staining scores, as evaluated by both the Oxford and SICCA grading systems. The mean Ocular Surface Disease Index (OSDI) score was 15.53 ± 13.13 in Group 1 and 15.92 ± 13.47 in Group 2 (*p* = 0.895). No significant differences were found in the average scores of the three OSDI subcategories. Numerical Rating Scale scores for postoperative discomfort were also not significantly different between groups.

### 3.3. CVFQ Outcomes and Driving-Related Discomfort

The Cataract Visual Function Questionnaire (CVFQ) results are summarized in Table 3. Overall scores and most subcategories did not differ significantly between the groups. However, Group 1 reported significantly less glare-related discomfort during driving.

**Figure 1:**
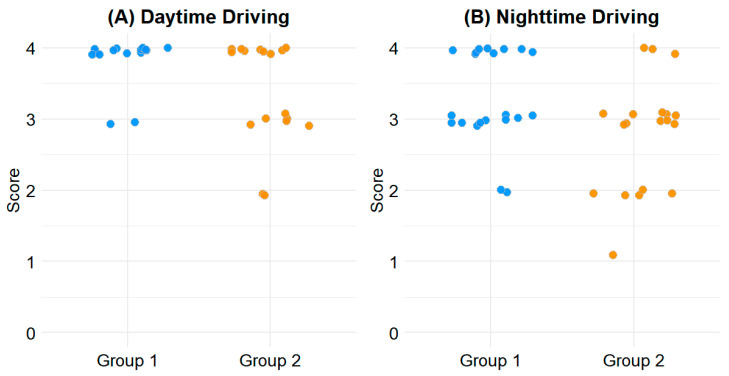
Scatterplot of driving-related CVFQ scores. (**A**) Daytime driving. (**B**) Nighttime driving. Y-axis indicates CVFQ scores ranging from 0 to 4, with higher scores indicating better visual comfort.

For daytime driving, the mean score in Group 1 was 3.93 ± 0.26, compared to 3.60 ± 0.65 in Group 2 (*p* = 0.020). For nighttime driving, Group 1 scored 3.48 ± 0.64, while Group 2 scored 3.00 ± 0.85 (*p* = 0.037). These differences indicate greater driving comfort in patients with less outdoor activity during the early postoperative period. The distribution of driving-related CVFQ scores is illustrated in Figure 1.

## 4. Discussion

This study compared postoperative visual function and patient satisfaction between individuals with differing levels of outdoor activity during the first month after cataract surgery. The results indicated that patients who engaged in less outdoor activity reported higher CVFQ scores for both daytime and nighttime driving, suggesting lower levels of visual discomfort. As driving difficulties are often a primary motivation for undergoing cataract surgery, the question of when patients can safely resume driving remains a key clinical concern [17].

Driving is a visually demanding task that differs from simpler daily activities in several critical aspects [18,19,20]. It requires the integration of multiple visual functions beyond standard visual acuity, such as contrast sensitivity, stereopsis, and peripheral visual fields, which are essential for detecting and responding to dynamic stimuli in real-world environments [18]. Furthermore, driving necessitates complex visuospatial processing and continuous focal adjustments across multiple distances, including intermediate and near vision, while also requiring rapid adaptation to changes in ambient lighting conditions [21,22]. These multifactorial visual demands suggest that full recovery of driving-related functions may require a longer adaptation period than other tasks [23]. Therefore, limiting exposure to variable environmental stimuli during the early postoperative period may assist in stabilizing visual function and reducing discomfort during driving. However, this study was limited in its ability to directly assess the aforementioned visual components, as the CVFQ does not include items specifically measuring stereopsis, peripheral fields, or contrast sensitivity. Additionally, contrast sensitivity testing was not performed, precluding objective confirmation of its role in driving-related outcomes.

Although the difference in driving-related CVFQ scores was statistically significant, both groups reported relatively high absolute scores (e.g., 3.93 vs. 3.60), suggesting that the clinical relevance of this finding may be limited. Though the age difference between groups was not statistically significant, the relatively younger mean age in Group 2 may have influenced both the level of outdoor activity and the pace of visual adaptation, potentially confounding the association between activity level and driving-related discomfort. Furthermore, other CVFQ subcategories did not differ significantly between groups, which warrants caution in interpreting the group difference in driving scores as indicative of overall postoperative satisfaction. In addition, the use of a single postoperative time point (1 month) may not have adequately captured longer-term trends in visual recovery and patient-reported outcomes.

It was hypothesized that higher levels of outdoor activity would be associated with greater ocular discomfort due to environmental exposure, leading to lower CVFQ scores. However, no significant differences were found between the two groups in overall CVFQ outcomes apart from the driving domain. Further analysis of dry eye-related parameters—including OSDI scores, tear breakup time, Oxford staining, and SICCA grading—also revealed no significant intergroup differences. Although previous studies have identified environmental factors such as humidity, air pollution, and temperature as contributors to dry eye exacerbation [24,25], the mere duration of outdoor activity may not sufficiently reflect the intensity or nature of environmental stressors. Moreover, all participants in this study received perioperative dry eye treatments, including cyclosporine, rebamipide, and corticosteroid eye drops, which may have mitigated ocular surface inflammation and improved tear stability [26,27]. These treatments likely reduced the impact of environmental exposure on dry eye symptoms, thereby attenuating differences in subjective discomfort between groups.

A key strength of this study is the use of the CVFQ, a culturally adapted and validated instrument for assessing postoperative visual function and patient satisfaction in the Korean population. Nevertheless, several limitations should be acknowledged. First, the outcomes were assessed only at one month postoperatively; longer follow-up periods are necessary to understand the trajectory of visual recovery and adaptation over time. Second, the average daily outdoor time was recorded solely based on patient recall without the use of objective measures, and the specific types of outdoor activities were not distinguished. Lastly, the relatively small sample size may have limited the statistical power to detect subtle differences in visual outcomes. In addition, as this study only involved patients with a single IOL type, the findings may not reflect outcomes associated with other IOLs. Future studies with larger cohorts and diverse IOL types are warranted to validate these findings and further explore the role of environmental and behavioral factors in postoperative visual adaptation.

## 5. Conclusions

Outdoor activity during the early postoperative period was not significantly associated with most CVFQ visual function scores, except for driving-related discomfort. While these findings provide useful insight, caution should be exercised when interpreting causality, given the small sample size; the use of perioperative eyedrops; and the absence of other visual parameters such as stereopsis, peripheral visual field, and contrast sensitivity. Larger-scale studies accounting for these factors are needed to validate the findings.

## Figures and Tables

**Table 1:** Patient demographics, and preoperative and postoperative biometric data.

Parameters	Outdoor Time ≤ 3 h/d (Group 1; *n* = 57)	Outdoor Time > 3 h/d (Group 2; *n* = 33)	*p* Value
Age (Years)	68.74 ± 12.74 (21–87)	64.15 ± 13.02 (34–82)	0.110
Gender (male/female)	19 (33.3%)/38 (66.7%)	16 (48.5%)/17 (51.5%)	0.231
Preoperative			
UDVA (LogMAR)	0.32 ± 0.22 (0.03–0.80)	0.33 ± 0.23 (0.00–0.70)	0.898
BCVA (LogMAR)	0.55 ± 0.25 (0.05–1.00)	0.47 ± 0.25 (0.01–0.80)	0.209
IOP (mmHg)	16.67 ± 2.05 (12–21)	17.36 ± 1.97 (15–21)	0.115
SE, manifest refraction (D)	−2.73 ± 5.83 (−27.00–5.50)	−2.93 ± 4.52 (−19.00–2.75)	0.302
Mean keratometry (D)	43.72 ± 2.02 (36.38–47.63)	43.81 ± 1.83 (38.00–46.25)	0.834
Postoperative			
UDVA (logMAR)	0.76 ± 0.19 (0.20–1.00)	0.77 ± 0.21 (0.40–1.20)	0.767
BCVA (logMAR)	0.92 ± 0.12 (0.70–1.00)	0.91 ± 0.11 (0.70–1.00)	0.593
IOP (mmHg)	14.98 ± 2.80 (10–21)	15.33 ± 2.31 (12–20)	0.524
SE, manifest refraction (D)	−0.68 ± 0.98 (−6.00–0.50)	−0.73 ± 0.74 (−2.50–1.00)	0.287
Mean keratometry (D)	43.86 ± 2.02 (36.88–47.75)	43.85 ± 1.87 (37.63–46.38)	0.981

Data are presented as mean ± SD (range: min–max) for continuous variables and as *n* (percentage) for categorical variables. D = diopters; UDVA = Uncorrected Distant Visual Acuity; BCVA = Best Corrected Visual Acuity; IOP = Intraocular Pressure; and MR = Manifest Refraction. *t*-tests were performed for age, preoperative UDVA, CDVA, IOP, mean keratometry, postoperative UDVA, IOP, and mean keratometry. Mann–Whitney U tests were used for preoperative SE, manifest refraction; and postoperative CDVA and SE, manifest refraction. The chi-square test was used for the categorical variable, gender.

**Table 2:** Postoperative assessments: tear breakup time, corneal staining score, Ocular Surface Disease Index, and Numerical Rating Scale at 1-month.

Parameters	Outdoor Time ≤ 3 h/d (Group 1; *n* = 57)	Outdoor Time > 3 h/d (Group 2; *n* = 33)	*p* Value
TBUT (sec)	4.63 ± 1.40 (2–8)	4.33 ± 1.80 (2–9)	0.452
Cornea staining score			
Oxford score (0–5)	0.46 ± 0.58 (0–2)	0.48 ± 0.85 (0–3)	0.534
SICCA score (0–3)	1.25 ± 1.38 (0–5)	1.30 ± 1.84 (0–7)	0.540
OSDI			
Physical symptoms score (0–4)	0.60 ± 0.55 (0.00–2.20)	0.76 ± 0.62 (0.00–2.20)	0.177
Discomfort during daily activities (0–4)	0.53 ± 0.67 (0.00–2.67)	0.45 ± 0.50 (0.00–2.00)	0.872
Aggravation by environmental factors (0–4)	0.78 ± 0.76 (0.00–2.67)	0.65 ± 0.83 (0.00–3.00)	0.259
Total OSDI score (25–100)	15.53 ± 13.13 (0.00–55.56)	15.92 ± 13.47 (0.00–56.25)	0.895
Numerical Rating Scale (0–10)	0.45 ± 1.08 (0–5)	0.50 ± 0.88 (0–3)	0.309

Data are presented as mean ± SD (range: min–max). TBUT = Tear Break-Up Time; SICCA = Sjögren’s International Collaborative Clinical Alliance; and OSDI = Ocular Surface Disease Index. *t*-tests were performed for TBUT and total OSDI score. Mann–Whitney U tests were used for Oxford score, SICCA score, and OSDI subcategory scores (physical symptoms, discomfort during daily activities, and aggravation by environmental factors).

**Table 3:** Postoperative assessments: Cataract-related Visual Function Questionnaire at 1-month.

Parameters	Outdoor Time ≤ 3 h/d (Group 1; *n* = 57)	Outdoor Time > 3 h/d (Group 2; *n* = 33)	*p* Value
CVFQ			
Overall visual quality (0–4)	3.28 ± 0.73 (2.00–4.00)	3.15 ± 0.67 (2.00–4.00)	0.345
Overall visual function (0–4)	3.53 ± 0.66 (1.00–4.00)	3.55 ± 0.56 (2.00–4.00)	0.931
Distance visual difficulties (0–4)	3.81 ± 0.30 (2.75–4.00)	3.78 ± 0.31 (3.00–4.00)	0.732
Near visual difficulties (0–4)	3.43 ± 0.60 (1.20–4.00)	3.38 ± 0.57 (2.00–4.00)	0.702
Glare symptoms			
Daytime driving (0–4)	3.93 ± 0.26 (3–4)	3.60 ± 0.65 (2–4)	0.020
Walking outside on a sunny day (0–4)	3.46 ± 0.76 (1–4)	3.44 ± 0.76 (1–4)	0.824
Night-time driving (0–4)	3.48 ± 0.64 (2–4)	3.00 ± 0.85 (1–4)	0.037
Looking at the street lights (0–4)	3.71 ± 0.46 (3–4)	3.57 ± 0.68 (2–4)	0.494
Total CVFQ score (25–100)	89.90 ± 8.52 (70.00–100.00)	86.38 ± 10.29 (65.75–100.00)	0.530

Data are presented as mean ± SD (range: min–max). CVFQ = Cataract-related Visual Function Questionnaire. *t*-tests were performed for near visual difficulties score and total CVFQ score. Mann–Whitney U tests were used for the remaining subcategories, including overall visual quality, overall visual function, distance visual difficulties, and glare symptoms (daytime driving, walking outside on a sunny day, night-time driving, and looking at streetlights).

## Data Availability

The datasets generated and analyzed during the current study are available from the corresponding author on reasonable request.

## Supplementary Materials

The following supporting information can be downloaded at https://www.mdpi.com/article/10.3390/diagnostics15131629/s1, Table S1: Overview of CVFQ: Items, subdomains.

## Abbreviations

The following abbreviations are used in this manuscript:

BCVA	Best Corrected Visual Acuity
CVFQ	Cataract-related Visual Function Questionnaire
OSDI	Ocular Surface Disease Index
SE	Spherical Equivalent
SICCA	Sjögren’s International Collaborative Clinical Alliance
TBUT	Tear Break-Up Time
UDVA	Uncorrected Distance Visual Acuity
IOP	Intraocular Pressure
NRS	Numerical Rating Scale
