# Supplementary material for: The Effects of Outdoor Activity on Patient-Reported Visual Outcomes Under Perioperative Management Using Cyclosporine and Rebamipide
Source: Diagnostics (Basel). 2025 Jun 26;15(13):1629. doi: 10.3390/diagnostics15131629 (PMC12248811; doi:10.3390/diagnostics15131629)
Supplement: Supplementary file 1 [file diagnostics-15-01629-s001.zip › diagnostics-3695554-supplementary.pdf]

Supplementary Table S1. Overview of CVFQ: items, subdomains.

Category/ Items	Answers				
Overall visual quality	Excellent	Good	Bad	Extremely poor	Cannot decide
Overall visual function	No difficulty	Some difficulty	Moderate difficulty	Very great difficulty	Cannot decide
Difficulty in daily activities – distance	No difficulty	Some difficulty	Moderate difficulty	Very great difficulty	Not applicable
3-1 Reading traffic signs or street signs					
3-2 Recognizing the faces of people					
3-3 Recognizing steps or stairs					
3-4 Watching television and reading subtitles					
Difficulty in daily activities–near	No difficulty	Some difficulty	Moderate difficulty	Very great difficulty	Not applicable
3-5 Using a computer					
3-6 Using a cellphone					
3-7 Reading a book or writing text					
3-8 Reading small print such as price labels in shops or labels on medicine bottles					
3-9 Doing fine handwork like clipping nails or sewing					
Glare	No difficulty	Some difficulty	Moderate difficulty	Very great difficulty	Not applicable
4-1 Daytime driving					
4-2 Walking outside on a sunny day					
4-3 Nighttime driving					
4-4 Looking at the street lights at night					
